# Invasive aspergillosis in critically ill hematology patients: outcomes and prognostic factors associated with mortality

**DOI:** 10.1186/cc9662

**Published:** 2011-03-11

**Authors:** G Burghi, V Lemiale, E Azoulay

**Affiliations:** 1 Hopital Saint-Louis, Université Paris VII, Paris, France

## Introduction

Invasive aspergillosis (IA) is documented in up to 15% of critically ill hematology patients admitted for acute respiratory failure. The disease is believed to be mostly deadly. Because diagnostic, preventive and therapeutic strategies for IA have changed over the past decade, we sought to appraise outcomes in hematology patients receiving mechanical ventilation for IA.

## Methods

Determinants of hospital mortality were identified in hematology patients admitted to the ICU for acute respiratory failure from proven or probable IA.

## Results

Fifty-nine patients received mechanical ventilation for IA over the 10-year study period. Thirty-six (62%) were neutropenic, 19 (32%) were receiving long-term steroids, and 13 (22%) were recipients of allogeneic BMT. Diagnosis was based on clinical and radiographic features, associated with either Aspergillus isolation (48 patients, including 25 bronchial aspiration, 17 BAL, six BAL + bronchial aspiration) or circulating galactomanan alone (11 patients). In 33 patients positive galactomanan was associated with Aspergillus isolation. Five cases were proven on autopsy. Associated bacterial infection was documented in 21 (35.6%) patients. Antifungal therapy included conventional amphotericin (50%), voriconazole (49%), liposomal amphotericin (32%), or caspofungin (19%). Seventeen (28.8%) patients had two lines of therapy and nine patients received a combination of voriconazole and caspofungin. Hospital mortality was 73% overall, 85% in patients with associated bacterial infection, and 44% in patients treated with voriconazole. Associated bacterial infection was independently associated with increased mortality (OR = 5.91 (1.04 to 33.5)), whereas the use of voriconazole (OR = 0.19 (0.04 to 0.91)) and localized disease (OR = 0.12 (0.03 to 0.59)) were associated with lower mortality.

## Conclusions

The use of mechanical ventilation in patients with IA complicating HM is associated with a high, yet not constant, mortality of 73%. Early management at a time where the disease is localized, as well as the use of voriconazole, translate into survival benefits.

